# Highly-excited Rydberg excitons in synthetic thin-film cuprous oxide

**DOI:** 10.1038/s41598-023-41465-y

**Published:** 2023-10-06

**Authors:** Jacob DeLange, Kinjol Barua, Anindya Sundar Paul, Hamid Ohadi, Val Zwiller, Stephan Steinhauer, Hadiseh Alaeian

**Affiliations:** 1 Department of Physics, Purdue University, West Lafayette, IN 47907 USA; 2 Elmore Family School of Electrical and Computer Engineering, Purdue University, West Lafayette, IN 47907 USA; 3 Department of Applied Physics, KTH Royal Institute of Technology, 106 91 Stockholm, Sweden; 4 School of Physics and Astronomy, University of St Andrews, St Andrews, KY16 9SS UK

**Keywords:** Single photons and quantum effects, Optical materials and structures, Nonlinear optics

## Abstract

Cuprous oxide ($\hbox {Cu}{}_2\hbox {O}$) has recently emerged as a promising material in solid-state quantum technology, specifically for its excitonic Rydberg states characterized by large principal quantum numbers (*n*). The significant wavefunction size of these highly-excited states (proportional to $n^2$) enables strong long-range dipole-dipole (proportional to $n^4$) and van der Waals interactions (proportional to $n^{11}$). Currently, the highest-lying Rydberg states are found in naturally occurring $\hbox {Cu}_2\hbox {O}$. However, for technological applications, the ability to grow high-quality synthetic samples is essential. The fabrication of thin-film $\hbox {Cu}{}_2\hbox {O}$ samples is of particular interest as they hold potential for observing extreme single-photon nonlinearities through the Rydberg blockade. Nevertheless, due to the susceptibility of high-lying states to charged impurities, growing synthetic samples of sufficient quality poses a substantial challenge. This study successfully demonstrates the CMOS-compatible synthesis of a $\hbox {Cu}{}_2\hbox {O}$ thin film on a transparent substrate that showcases Rydberg excitons up to $n = 8$ which is readily suitable for photonic device fabrications. These findings mark a significant advancement towards the realization of scalable and on-chip integrable Rydberg quantum technologies.

## Introduction

Photons are highly promising resources for leveraging the principles of quantum mechanics during the second quantum revolution. Unlike particles such as atoms or ions, photons are less susceptible to environmental disturbances and can be generated, measured, controlled, and transmitted over long distances at the ultimate speed. However, the negligible interactions between photons in linear media present a significant obstacle to the advancement of scalable quantum networks and technologies^1–3^. The development of single-photon nonlinearities would effectively overcome this challenge and expedite progress in optical quantum technology. Crucial to achieving these nonlinearities are matter-based systems that engage with photons through robust, long-range dipole-dipole interactions. For instance, the utilization of organic dopants within crystal lattices has successfully demonstrated nonlinear effects encompassing single-photon optical switching and on-demand single photon generation^4–7^, accompanied by other notable quantum phenomena like photon storage^8,9^.

An extensively explored avenue for achieving single-photon nonlinearities lies within the realm of Rydberg systems. Rydberg states, characterized by high principal quantum numbers, *n*, play a pivotal role in facilitating interactions between photons and generating substantial optical nonlinearities. These states exhibit long lifetimes, proportional to $n^3$, and possess wavefunction sizes that scale as $n^2$. Notably, the latter property enables Rydberg-Rydberg interactions through long-range dipolar and van der Waals interactions, which scale as $n^4$ and $n^{11}$, respectively^10,11^. Such strong, long-range interactions among Rydberg states result in the phenomenon known as the *Rydberg blockade* wherein the existence of one excitation inhibits the formation of another excitation within a defined *blockade radius*. This phenomenon forms the basis of a wide array of recent breakthroughs in atomic Rydberg quantum systems, including quantum few-body photonic states^12–14^, all-optical transistors^15,16^, high-fidelity two-qubit gates^17,18^, and analog quantum simulators^19–23^.

Excitons, analogous to atoms in solid-state materials, consist of a positively-charged hole and a negatively-charged electron, giving rise to energy levels like those of hydrogen atoms, which can exhibit Rydberg states, as well^24^. Unlike atoms, however, excitons are embedded in solid-state hosts, which opens up exciting possibilities for quantum devices by combining the extraordinary properties of Rydberg states with inherent durability, compactness, and scalability. The energy levels of excitons follow the Rydberg formula^25^ as

1 $$\begin{aligned} E_n = E_g - \frac{Ry}{(n - \delta _n)^2} , \end{aligned}$$

where $E_g$ is the bandgap energy, *Ry* is the binding energy, *n* is the principal quantum number of the state, and $\delta _n$ is the quantum defect, which describes perturbations caused by the screening of Coulombic interactions within solid-state lattices and the non-parabolicity of the band structure^24^.

**Figure 1:**
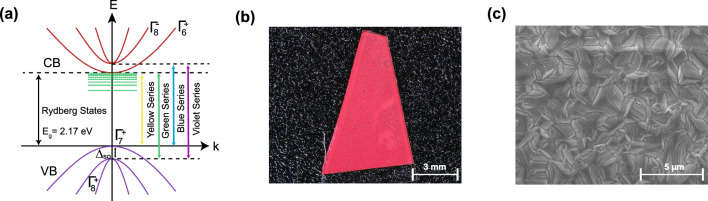
Overview of synthetic $\hbox {Cu}{}_2\hbox {O}$ sample. (**a**) Band structure of $\hbox {Cu}{}_2\hbox {O}$ showing all Rydberg series. (**b**) Microscope and (**c**) scanning electron microscope, image of the synthetic sample.

In recent studies, cuprous oxide has emerged as a highly favorable semiconductor host for Rydberg excitons^24^, offering the necessary properties to establish the blockade effect within a solid-state platform^26–28^. Illustrated in Fig. 1a, cuprous oxide exhibits a direct bandgap ($E_g \approx 2.17$ eV) and possesses a symmetric cubic lattice structure that effectively suppresses phonon coupling. Furthermore, it boasts a significant binding energy (approximately 98 meV), enabling the accommodation of a substantial number of Rydberg states without thermal ionization^24^. Transitions between various valence and conduction bands result in the formation of four exciton series, characterized by the colors of the emitted photons, namely the yellow, green, blue, and violet Rydberg series. Of particular interest is the yellow series, where the valence band shares the same parity as the conduction band. This parity selection renders p-excitons dipole-allowed for direct recombination to the valence band, while 1s-excitons are dipole-forbidden, resulting in an extended lifetime of up to 1 $\upmu \hbox {s}$^24,29^. This exceptional property sets cuprous oxide ($\hbox {Cu}{}_2\hbox {O}$) apart from other materials such as transition metal dichalcogenides (TMDs), which possess enormous binding energies^30,31^ but have short-lived ground states due to the presence of dipole-allowed states^32^.

To date, the majority of investigations about Rydberg excitons in Cu_2_O have concentrated on bulk samples acquired from the Tsumeb mine in Namibia, which were subjected to mechanical polishing to ensure surface smoothness^33–39^. These studies have documented states with principal quantum numbers up to $n = 30$^37^. However, for future technological applications to thrive, the attainment of high-quality synthetic $\hbox {Cu}_2\hbox {O}$ is paramount. Consequently, recent endeavors have been made to cultivate cuprous oxide films featuring highly-excited Rydberg excitons^40–42^. These efforts not only hold the promise of scaling $\hbox {Cu}_2\hbox {O}$ but also enable the coupling of excitons to on-chip nanophotonic circuits. Furthermore, in thin-film samples with thicknesses smaller than the blockade radius, an extraordinary nonlinearity at the level of a single photon can be obtained, a long-sought property that can be harnessed in quantum devices like single-photon sources or low-intensity optical switches, as well as many-body quantum optical studies^26,43,44^.

In this paper, we introduce the first spectroscopic observation of Rydberg excitons in a synthetic $\hbox {Cu}_2\hbox {O}$ thin film on a transparent substrate, $\hbox {SiO}_2$^45^, and present the first use of the Bayesian reconstruction technique on data from a synthetic $\hbox {Cu}_2\hbox {O}$ sample and demonstrate its use to verify the presence of high-energy excitons. Further, we delve into an examination of the influences of temperature and excitation power on these states. Our study paves the way for seamless, monolithic integration of Rydberg excitons with photonic devices, thanks to the technique’s CMOS-compatibility and the substrate’s transparency within the wavelength range of Rydberg excitons.

## Results and discussions

To synthesize the sample, a 700 nm Cu film was deposited on the substrate via e-beam evaporation, with a 5 nm Ti layer in between the Cu and the substrate to increase adhesion. The Cu layer was oxidized in synthetic air for several hours to ensure its full oxidation^41^. Figure 1b,c show a microscope image of the finished sample as well as a scanning electron microscope (SEM) image, respectively.

### Optical density measurement

**Figure 2:**
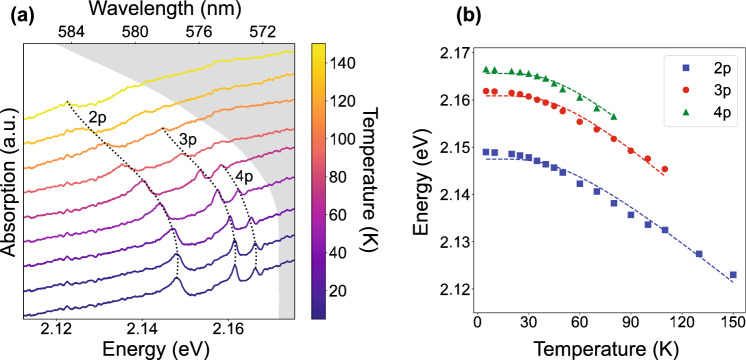
Results from optical density measurement. (**a**) Temperature-dependent OD of the synthetic $\hbox {Cu}_2\hbox {O}$ film. Line colors correspond to different temperatures. The grayed-out region represents energies above the bandgap energy, determined using Elliott’s model, see Eq. (2). (**b**) Rydberg exciton 2p, 3p, and 4p peaks as a function of temperature. The dashed lines represent the least squares fit from Elliott’s model.

As the sample is on a transparent substrate, Rydberg excitons can be examined through optical density (OD) measurements. The excitation light from a broadband white light source (Thorlabs SLS201L) was focused onto the sample with an objective lens with NA = 0.42 (Mitutoyo Plan Apo 20x). The transmitted light was collected with a similar objective and sent to a spectrometer (Princeton Instruments HRS-750, 1200 gratings/mm, 50 $\upmu \hbox {m}$ slit width, resolution of 0.036 nm). The measurement was repeated at temperatures from 5–150 K with a precision of 0.25 K. Figure 2a shows the results from this measurement for the yellow exciton series. At low temperatures, resonances up to $n=4$ were observed. As the temperature was increased, the exciton resonances were red-shifted and broadened until they could no longer be resolved. At temperatures higher than 150 K, no distinct exciton resonances could be observed. The energy shift can be attributed to changes in the bandgap energy, binding energy, and quantum defects of $\hbox {Cu}_2\hbox {O}$ as a function of temperature. The change in the bandgap energy, $E_g$, arises from the thermal expansion of the crystal lattice and phonon-electron interactions^46^. The binding energy, *Ry*, being proportional to the reduced mass of the exciton, is also temperature-dependent due to changes in the electronic bands’ curvatures with the temperature^24^. Finally, the quantum defect, which arises in part due to the non-parabolic bands, will likewise vary with temperature as the band structures are modified^47^. In the remainder of this section, we present the first analysis of temperature-dependent exciton behavior in synthetic $\hbox {Cu}_2\hbox {O}$ and compare it to previous results on natural samples^36^.

The changes in the first two parameters can be summarized by Elliott’s model (2), which assumes that shifts are dominated by continuous absorption from $\Gamma _3^-$ phonons^46^,

2 $$\begin{aligned} \begin{aligned} E_g(T) = E_{g0} + E_{gT} \Bigg [\coth {\Bigg (\frac{\hbar \omega _3}{2 k_B T}\Bigg )} - 1\Bigg ] ,\\ Ry(T) = Ry_{0} + Ry_{T} \Bigg [\coth {\Bigg (\frac{\hbar \omega _3}{2 k_B T}\Bigg )} - 1\Bigg ] , \end{aligned} \end{aligned}$$

with $$\ \hbar \omega _3 = 13.6$$ meV.

$E_{g0}$ and $Ry_0$ are temperature-independent terms that represent the low-temperature limit of the bandgap and binding energies, respectively, and $E_{gT}$ and $Ry_T$ capture the temperature effects. To fit this model to our data, the Rydberg energies were extracted for each temperature using least squares fitting to an asymmetric Fano lineshape^48^

3 $$\begin{aligned} \alpha _n(E) = f_n \frac{\frac{\Gamma _n}{2} + 2 q_n (E - E_n)}{\big (\frac{\Gamma _n}{2}\big )^2 + (E - E_n)^2} , \end{aligned}$$

where $E_n$ is the *n*th exciton energy, $\Gamma _n$ is the corresponding linewidth, $f_n$ is proportional to the oscillator strength, and $q_n$ is an asymmetry factor modeling the interference between narrow optical transitions and the phonon continuum^49^.

As is visible in Fig. 2a, there is a background from continuum absorption which increases at higher energies. This background, known as the Urbach tail, signifies an increase in absorption near the bandgap energy caused by an increasing density of states in that energy range. This increase is described by an exponential function,

4 $$\begin{aligned} \alpha _{U}(E) = \alpha _0 \exp {\Bigg (\frac{E - E_g}{E_u}\Bigg )} , \end{aligned}$$

where $E_g$ is the bandgap energy, $\alpha _0$ is the magnitude of the continuum absorption, and $E_u$ is the Urbach energy^50,51^.

Elliott’s model was used to fit the center energy as a function of temperature, taking into account the 2p, 3p, and 4p peaks simultaneously, as shown in Fig. 2b. For simplicity, we ignored the quantum defects for all three resonances at all temperatures. This is just an approximation since the quantum defects vary with *n* and are expected to be temperature-dependent as well. Typical methods to extract the quantum defect as a function of *n*, such as those used in^36^, require the observation of high energy peaks whose quantum defects approach a constant value (see Sect. S3 of the supplementary materials for more details). Theoretical calculations show that this trend does not emerge until $n \gtrapprox 10$^47^. However, in^36^ the authors note that while assuming $\delta _n(T) = 0$ is simplistic, it yields results that agree with both the literature and more detailed analyses which include the quantum defect corrections. From this fit, we have extracted the parameters shown in Table 1.

**Table 1:** Fit parameters from Elliott model.

$E_{g0}$ (meV)	$E_{gT}$ (meV)	$Ry_0$ (meV)	$Ry_T$ (meV)
2171.7 ± 0.04	− 29.5 ± 1.75	96.8 ± 2.12	− 20.9 ± 8.09

The values of $E_{g0}$, $E_{gT}$, and $Ry_0$ are in agreement with the literature, but the extracted $Ry_T$ is different^33,36^. It must be noted that ignoring $\delta _n$ is valid for describing the temperature dependence of the bandgap energy but breaks down for the binding energy, so we attribute this particular discrepancy to the errors caused by this assumption. Overall, the accurate observations of $E_{g0}$, $E_{gT}$, and $Ry_0$ confirm that the observed absorption lines indeed arise from Rydberg excitons in the synthetic $\hbox {Cu}_2\hbox {O}$.

### Photoluminescence measurement

Non-resonant photoluminescence (PL) measurements were performed with a 532 nm laser to excite electrons above the bandgap and create free electrons which form bound states as they relax to the lower exciton levels. The laser was focused down to a spot size of $\hbox {FWHM}=3.1$ $\upmu \hbox {m}$ using an objective lens (Mitutoyo Plan Apo 20x). The PL from the excitons was collected with the same objective and sent to the spectrometer after a long-pass filter (Semrock LP03-532RE-25) and a dichroic mirror (Thorlabs DMLP567) were used to block the reflected pump laser. Temperature-dependent data was taken at an incident laser power of 50 $\upmu \hbox {W}$ at temperatures from 5 to 150 K. Power-dependent data was taken with the sample held at a constant temperature of 5 K at laser powers ranging from 50 $\upmu \hbox {W}$ to 2 mW.

**Figure 3:**
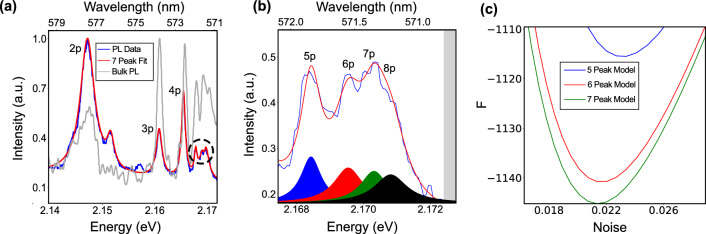
Yellow exciton PL spectrum at a temperature of 20 K. (**a**) View of entire yellow exciton spectrum with 7-peak fit extracted from Bayesian analysis overlaid in red. The gray reference line represents a PL spectrum acquired from a bulk spectrum at low temperature. (**b**) Zoomed-in view of the region encompassed by the black dashed circle in (**a**). Deconvolved 5p, 6p, 7p, and 8p lineshapes (scaled to fit the panel) are overlaid at the bottom of the graph. The light gray region represents energies above the bandgap. (**c**) The plot of F vs. noise level from PL spectrum Bayesian analysis indicates that the 7-peak model (which fits resonances up to 8p) is the most probable given this dataset.

Figure 3a,b show the PL spectrum at the brightest point on the sample at a temperature of 20 K. Rydberg states up to 7p are clearly visible, but it is visually apparent if there are contributions from higher-lying states with weaker transition moments. Using Bayesian analysis, we have verified the presence of the $n = 8$ state as well. Proof of this is shown in Fig. 3c, which compares the effective free energy, *F*, for a 5-peak, 6-peak, and 7-peak fit of the data (cf. “Methods” for a detailed explanation).

The higher number of observed Rydberg states in the PL measurement compared to the OD measurement can be attributed to the spatial non-uniformity of the sample and the broader spot size of the excitation. Another curious feature of the PL spectrum is the deviation of peak heights from the expected $n^{-3}$ trend, which is made evident by comparison to the reference spectrum acquired from a bulk sample (cf. gray line in Fig. 3a). This deviation could arise from a variety of sources, such as the collisional population of Rydberg states during non-resonant PL excitation, which may not follow the standard scaling law, as well as scatterings from vacancies, the presence of the green 1S exciton, which can influence the matrix elements at lower *n* values, and the susceptibility of higher-*n* states to the effects of charge impurities and local electric fields^52^.

**Figure 4:**
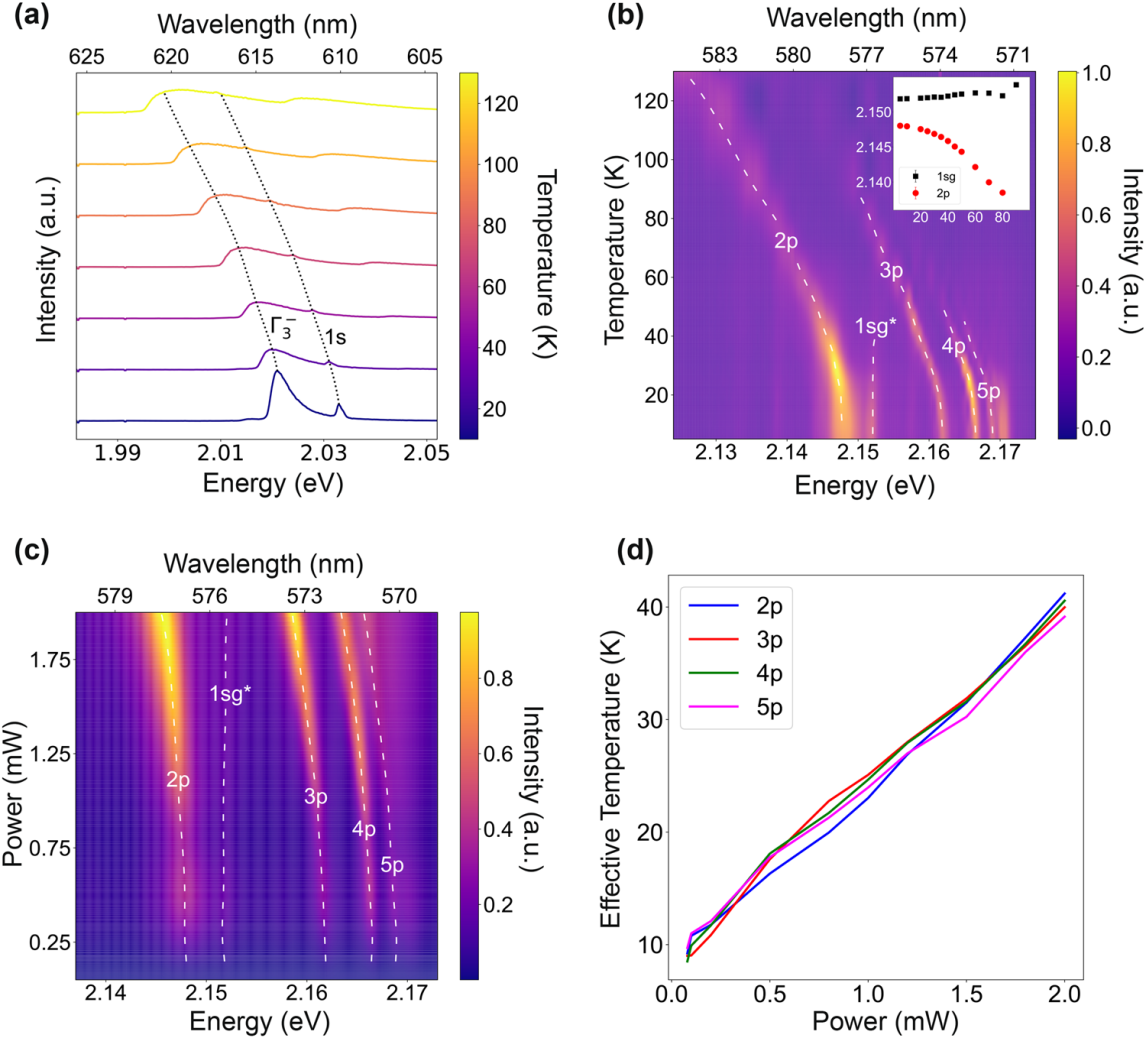
Summary of results from PL measurement. (**a**) Temperature varying PL from $\hbox {1S}_y$ and phonon replica region. (**b**) Temperature varying PL from yellow exciton series. The line labeled as “1s${}_{\text {g}}^{*}$” has often been attributed to the green 1s resonance. (**c**) Power dependent PL spectrum from synthetic $\hbox {Cu}{}_2\hbox {O}$ sample. (**d**) Interpolation of effective temperature induced by heating from 532 nm pumping laser.

Figure 4a shows the results from the phonon replica region of the spectrum, where the quadrupole-allowed and $\Gamma _3^-$ phonon-assisted relaxations of the yellow 1s-orthoexciton state can be observed as Fano and Boltzmann-tailed peaks, respectively. At higher temperatures, one can see an anti-stokes phonon-assisted transition appearing at higher energies. As indicated by the black dotted line, these peaks red shift with temperature similar to the trend obtained from the OD measurement. The information from this spectral range is also useful for gauging the prevalence of metallic impurities in the sample. Excitons bound to these impurities fluoresce at energies between 1.99 and 2.01 eV, with intensities on the same order of magnitude as the $\Gamma _3^-$ phonon-assisted transition^53^. As can be seen in Fig. 4a, such features are absent from the spectrum, even at low temperatures, demonstrating that the synthetic film is metallic impurity-free.

For the temperature and power-dependent analyses, the peaks were fit with Fano lineshapes using a least squares algorithm. Figure 4b shows the results from the temperature-dependent study. As in the OD measurements, the energy of the yellow exciton states observed in PL redshift as temperature increases. A similar trend can be observed in the power-dependent data shown in Fig. 4c. The change in the Rydberg energies can be attributed to two possible effects: temperature change due to laser absorption and exciton–exciton interactions caused by the high exciton density created at high laser powers. As already discussed in “Optical density measurement”, the former effect manifests as a redshift, while the latter is expected to manifest as a blueshift, caused by repulsive van der Waals interactions^11^.

To differentiate between these two effects, we performed an interpolation, using the center energies from the power-dependent measurement to extract an effective temperature as a function of the laser power. Separate interpolations were performed for the 2p, 3p, 4p, and 5p peaks, with the most contributions even at elevated temperatures. As can be seen in Fig. 4d, all four interpolations follow the same trend. In the presence of notable exciton–exciton interactions, however, one would anticipate that the interpolations from higher states deviate from the lower ones. This is because excitons in higher energy states exhibit stronger interactions, attributed to the overlap of their extended wavefunctions. Combined with the linear trend, this indicates that the energy shifts due to temperature change dominated over those from the exciton–exciton interaction, due to the strong above-the-bandgap absorption.

**Figure 5:**
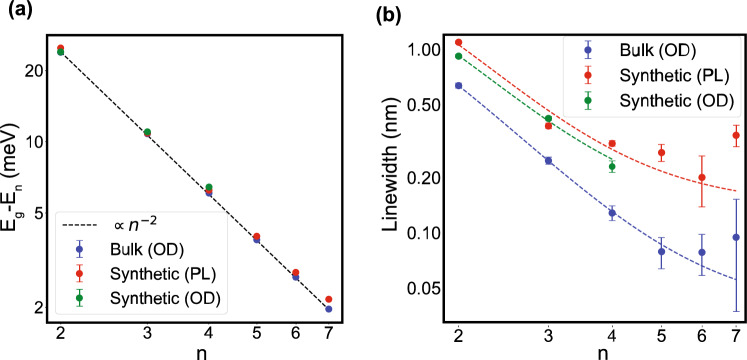
Trends of various resonance parameters vs. the principal quantum number. (**a**) Center energy as a function of *n*, with an $n^{-2}$ trendline overlaid. (**b**) Linewidth as a function of *n*. The dashed lines represent fits of the model given in Eq. (5) for the data series of the same color.

In Fig. 5a,b, we show the energy and linewidth of Rydberg excitons, respectively, comparing the synthetic and natural $\hbox {Cu}{}_2\hbox {O}$ samples. These properties are plotted as a function of *n* for data obtained from PL and OD measurements of the synthetic sample at the lowest temperature of 5 K and the lowest laser power of 50 $\upmu \hbox {W}$, as well as for data from OD measurements of a natural bulk sample (see Sect. S3 of the supplementary materials).

The black dashed line in Fig. 5a shows the ideal $n^{-2}$ trend of the Hydrogenic Rydberg series. As can be seen, the Rydberg energies of the synthetic film and the natural bulk sample follow this trend, indicating that confinement effects are negligible for these states. (cf. Sect. S1 of the supplementary material).

As for the linewidths depicted in Fig. 5b, for ideal Rydberg states a $n^{-3}$ scaling is expected, but as can be observed, here they reach a plateau which agrees with previous results^33^. The *n*-dependent behavior can be modeled as

5 $$\begin{aligned} \Gamma (n) = \alpha \frac{n^2 - 1}{n^5} + \beta , \end{aligned}$$

where $\alpha$ is a proportionality constant and $\beta$ represents a minimum value which is the linewidths for very large *n*^54^. This model was used to fit the data from each series shown in Fig. 5b. The high-*n* asymptotes ($\beta$) for each series are reported in Table 2. A similar linewidth plateau has been observed in ultra-cold Rydberg atoms interacting with a dense background gas^55^, a phenomenon attributed to the frequent scattering of high-lying Rydberg electrons off of the ground-state atoms^56^. By analogy with the atomic case, we hypothesize a similar phenomenon could affect Rydberg excitons in $\hbox {Cu}{}_2\hbox {O}$, resulting from collisions with a background electron-hole plasma, phonons, or a high-density of 1s ground-state excitons^57,58^, which will be reported elsewhere.

**Table 2:** Broadening linewidths, given by $\beta$ in Eq. (5), for various measurements of Rydberg excitons in $\hbox {Cu}{}_2\hbox {O}$ (nm).

Bulk (OD)	Synthetic (PL)	Synthetic (OD)
0.061 ± 0.009	0.181 ± 0.029	0.181 ± 0.043

## Conclusion

In this study, we have conducted optical spectroscopy measurements to investigate Rydberg excitons in a synthetic, thin-film sample of $\hbox {Cu}{}_2\hbox {O}$ grown on a transparent substrate using CMOS-compatible techniques. Our findings reveal the presence of yellow exciton Rydberg states up to $n = 4$ and $n = 8$ as determined by OD and PL measurements, respectively. Furthermore, we have explored the temperature-dependent behavior of these excitons, which aligns well with Elliott’s model, consistent with previous reports on natural bulk $\hbox {Cu}{}_2\hbox {O}$ crystals^36^. Additionally, we have observed spectral variations induced by changes in excitation power, which can be adequately explained by heating effects resulting from optical absorption.

By establishing a solid-state quantum platform centered around Rydberg states, this study lays a crucial foundation. The blockade effect has been observed in bulk $\hbox {Cu}{}_2\hbox {O}$ samples recently^59^ and achieving this effect in synthetic crystals would mark a substantial advancement toward leveraging the potential of this unique feature for on-chip quantum technologies. As a result, the immediate next step after this study is to enhance the sample quality and crystallinity, enabling access to higher-*n* states and facilitating the observation of Rydberg blockade effects in both spectral and temporal dynamics.

The field of semiconductor Rydberg physics is still in its nascent stage, and our successful demonstration of Rydberg excitons in synthetic $\hbox {Cu}{}_2\hbox {O}$ opens up new avenues for further exploration in more intricate environments. This includes investigating nanophotonic circuits capable of introducing significant optical nonlinearities^41,60^, as well as optical cavities that facilitate the strong interaction of Rydberg exciton-polaritons^39^. These advancements have the potential for disruptive Rydberg technologies such as photonic quantum gates, on-demand single-photon and quantum light sources^43,44^.

## Methods

### Bayesian reconstruction of optical density measurement on bulk $\hbox {Cu}{}_2\hbox {O}$

As has been established, high energy exciton peaks become hard to distinguish because their oscillator strengths decrease as $n^{-3}$ and their spacings decrease as $n^{-2}$, causing the peaks to overlap as they approach the bandgap^24^. Because of this, least squares fitting algorithms are not adequate for discerning high energy exciton resonances (this is why we studied only resonances with $n \le 7$ in the previous sections). A more effective fitting can be done with Bayesian reconstruction^61^, which helps to identify even those features which have intensities below the noise level.

In Bayesian reconstruction, a model with fit parameters $\theta$ can be fitted to a dataset *D* by leveraging Bayes’ law

6 $$\begin{aligned} P(\theta |D)\propto P(D|\theta )P(\theta ) , \end{aligned}$$

where $P(\theta |D)$ represents the probability of a given set of fit parameters given the measured dataset, $P(\theta )$ is the prior probability of the fit parameters, and $P(D|\theta )$ is the probability of a dataset being measured given an underlying set of fit parameters, described by the equation

7 $$\begin{aligned} P(D|\theta ) \propto \exp \Bigg [-\frac{n\epsilon (\theta )}{\sigma ^2}\Bigg ] , \end{aligned}$$

where $\sigma$ is the standard deviation of the background noise, assumed to be Gaussian, and $\epsilon (\theta )$ is the mean-squared error (MSE) between the dataset and the model. The *Metropolis-Hastings algorithm*, as described in^62^, can be used to sample from $P(D|\theta )$ and estimate the underlying distribution of the fit parameters. However, this method requires knowledge of the system's underlying noise level, which cannot be known in advance. Furthermore, it provides no benchmark by which to compare different models, which is essential for determining how many peaks are present in a spectrum. To resolve this issue, we used the *Replica Exchange Monte Carlo (RXMC) method*, which generalizes the Metropolis-Hastings algorithm to test multiple potential noise levels simultaneously and contrast the likelihoods of different models^63^. The output of this algorithm is a parameter *F*, defined as^63^

8 $$\begin{aligned} F = -\log {P(D|K,\sigma )}, \end{aligned}$$

where *P*(*D*|*K*, *b*) is the probability of a dataset being measured given that the underlying system follows some model *K* and has a background noise $\sigma$. *F* is the weighted average of the dataset’s probability of being generated by a given set of fit parameters over the distribution of fit parameters found by the algorithm^61^. It is analogous to the Helmholtz free energy, which, in statistical mechanics, is minimized by the most probable configuration. Similarly, the *F* found by the RXMC method is minimized by the most probable model and background noise^63^. A unique value of *F* is given for each background noise for each model tested. The model with the lowest minimum *F* is deemed the most probable.

### Spectral decomposition of bulk $\hbox {Cu}{}_2\hbox {O}$ using Bayesian estimation

**Figure 6:**
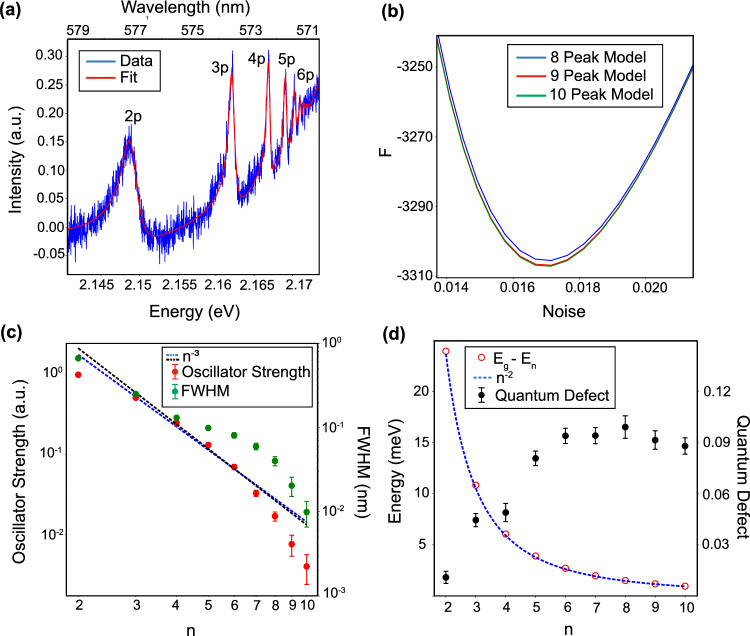
Overview of Bayesian reconstruction from bulk $\hbox {Cu}{}_2\hbox {O}$ sample. (**a**) Raw and fitted absorption spectrum showing 2–6p excitonic resonances. (**b**) *F* vs. noise level for 8–10 peak models. (**c**) Oscillator strengths and spectral full widths at half maximum (FWHM) from the ten peak model compared to an $n^{-3}$ trendline. (**d**) Rydberg exciton resonance energies and quantum defects from the ten peak model. The blue dotted curve is a trendline showing $n^{-2}$ dependence.

The spectroscopic absorption data set *D* was measured from a bulk natural $\hbox {Cu}{}_2\hbox {O}$ crystal sandwiched between two $\hbox {CaF}{}_2$ windows. Figure 6a shows the raw and fitted absorption spectra where the 2–6p exciton peaks are clearly distinguishable. More peaks are visible, but their spectral overlap makes it difficult to investigate them with certainty. To resolve this issue, Bayesian reconstruction was used. Multiple models (corresponding to different numbers of peaks) were fit to the same dataset using the RXMC method. Each peak was fit with a Fano function, and the exponential behavior near the bandgap of $\hbox {Cu}{}_2\hbox {O}$ was modeled by an Urbach tail. The center energies of each peak were determined from the bandgap energy ($E_g$), the binding energy (*Ry*), and the quantum defects ($\delta _n$), which were all left as fit parameters.

Figure 6b shows the plots of *F* as a function of background noise for each model. As can be seen, the ten peak model gives the lowest value for *F*, indicating that there are ten distinct resonances contributing to the absorption spectrum shown in Fig. 6a. Here, Bayesian reconstruction allows us to determine the presence of high-energy peaks despite the fact that they cannot be distinguished visibly because of their intensity falling below the noise floor.

To test the validity of these results, we plotted the values of the oscillator strengths, linewidths, resonance energies, and quantum defects as functions of *n*. Figure 6c shows the oscillator strengths and spectral linewidths of the ten-peak model. The oscillator strengths decrease as $n^{-3}$ as shown by the dotted line. However, the linewidths do not. As discussed in the main text, we speculate that this broadening may be caused by interactions with a background of electron-hole plasma. Figure 6d shows the energies of the exciton resonances, which follow an  $n^{-2}$ dependence as expected for Rydberg levels. It also shows the *n*-dependent quantum defects, which start to saturate around $n = 6$, a behavior consistent with previous theoretical studies^47^. For more details, the complete list of fit parameters for the ten-peak model is given in the supplementary material S1.

### Supplementary Information

Supplementary Information.

## Data Availability

The data presented in the paper is available at https://doi.org/10.5281/zenodo.7277624.

## Code availability

The Bayesian analysis code is available at https://github.com/PurdueQNP/Bayesian-Analysis. Other code used in the data analysis presented here is available from the authors upon reasonable request.
