# Supplementary material for: Highly-excited Rydberg excitons in synthetic thin-film cuprous oxide
Source: Sci Rep. 2023 Oct 6;13:16881. doi: 10.1038/s41598-023-41465-y (PMC10558487; doi:10.1038/s41598-023-41465-y)
Supplement: Supplementary file 1 — Supplementary Information. [file 41598_2023_41465_MOESM1_ESM.pdf]

Supplement: Highly-Excited Rydberg Excitons in Synthetic Thin-Film Cuprous Oxide

I. COMPARISON OF EXCITON SIZE AND BLOCKADE RADIUS TO SAMPLE THICKNESS

As mentioned in the main text, excitons may experience confinement effects when their wavefunctions are comparable to the Cu_2O film thickness [1]. The p-series exciton wavefunction size can be estimated as [2]

$$r_n = a_b(3n^2 - 2)\,, \tag{1}$$

where a_b is the Bohr radius of the yellow excitons with a value of 1.11 nm [3]. Thus, even for the 8p state, the highest state observed in this work, the wavefunction is only about 210 nm large and thus would be too small to experience noticeable perturbations from the confinement. This is verified in Fig. 5(a) in the main text, where all data points closely follow the n^{-2} trendline. Despite this, it should be noted that even though individual excitons may be too small to experience confinement effects, inter-exciton interactions may still be affected. The blue data points in Fig. 1 represents the blockade radius as a function of the principal quantum number, which can be approximated by the relation [4]

$$r_{\text{blockade}} = 4\text{ nm }n^{7/3}. \tag{2}$$

As can be seen, the high principal quantum number states (in particular $n = 8$), have blockade radii approaching the thickness of the sample studied here, raising the possibility of observing the desired blockade effect.

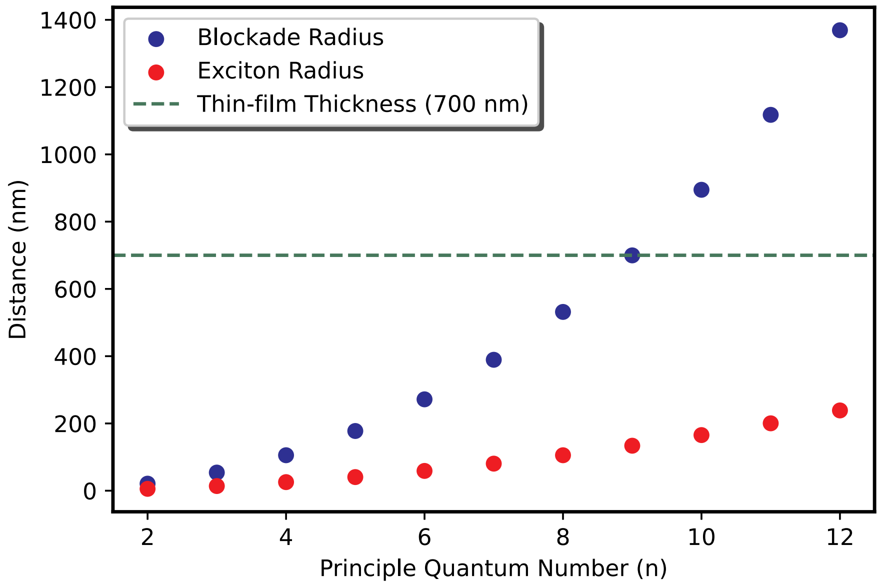

FIG. 1. Plot of exciton size (red data) and blockade radius (blue data) as a function of principal quantum number. The green dashed line represents the sample thickness of 700 nm.

II. PHOTOLUMINESCENCE MAP OF SAMPLE SURFACE

Since the PL of the 1s yellow orthoexciton is several orders of magnitude higher than the PL from the yellow exciton series [5], we used it to examine the homogeneity of the sample and find the most optically active region of the sample. Using a nano-positioner we scanned the sample through an 1800 × 2000 μm region in increments of 400 μm, recording the brightness of the Γ_3^- and 1s-orthoexciton peaks at each position. Once the brightest spot was determined via

this coarse analysis (with the brightest spot being roughly twice as bright as the dimmest), a more fine analysis was conducted by scanning over a 10 × 10 μm region in increments of 1 μm. Whereas the coarse analysis yielded substantial variation in the brightness of these peaks, the fine analysis yielded no more than 5% change. The results of the coarse analysis are shown in Fig. 2. Panel (a) shows how the peak height of the 1s yellow ortho-exciton peak varies with position while panel (b) shows how the background in the phonon replica region of the spectrum varies with position. As can be seen, both vary substantially. This could arise from a number of factors including scattering from the sample's rough, microcrystalline surface as well as strain distribution since the sample is not lattice-matched to its substrate. Currently, there are no studies that analyze how these factors affect exciton behavior. In future work, these trends can be studied more carefully by fabricating samples that systematically control these parameters.

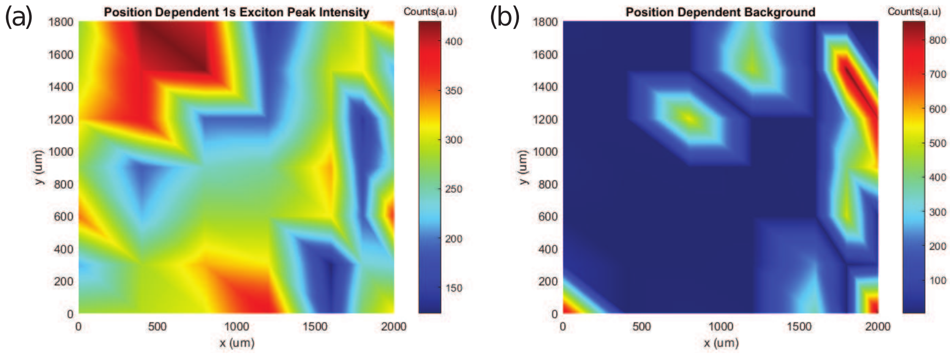

FIG. 2. Variation of exciton spectrum as a function of position on the sample. (a) Plot of 1s yellow orthoexciton peak height as a function of position on the sample. (b) Contour plot of background counts in the phonon replica region as a function of position.

III. CALCULATION OF SPECTRAL FITTING PARAMETERS

As mentioned in the main text, the 10-peak model was the most probable fit for the bulk Cu_2O data. The best-fit parameters from the model are given in Tables I and II. Table I gives the peak-independent parameters, including the bandgap energy of the system (E_g), the Rydberg binding energy (Ry), the inhomogeneous broadening (σ), the Urbach absorption coefficient (a_0), and Urbach energy (E_u). Table II gives the peak-dependent parameters, namely the quantum defects (δ_n), spectral linewidths (Γ_n), Fano asymmetry factors (q_n), and peak heights (f_n), for each peak.

TABLE I. Peak-independent parameters from Bayesian reconstruction of bulk Cu_2O OD spectrum

E_g(eV)	R_y(meV)	σ (nm)	α_0 (unitless)	E_u (eV)
2.173	94.9	0.02	0.2864	0.008

TABLE II. Peak-dependent parameters from Bayesian reconstruction of bulk Cu_2O OD spectrum.

n	δ_n	Γ_n (nm)	q_n	f_n (a.u.)
2	0.0096	0.66	3.23	0.185
3	0.043	0.249	4.51	0.0956
4	0.0436	0.129	3.64	0.0458
5	0.0789	0.0992	5.469	0.0246
6	0.0942	0.0787	9.1153	0.0127
7	0.0919	0.0611	9.1415	0.006
8	0.0986	0.0414	9.359	0.003
9	0.0907	0.0217	7.4665	0.0014
10	0.0909	0.0103	7.2898	0.00076
11	0.09	0.0051	6.666	0.00014

Tables IV and III give the peak-dependent and peak-independent parameters from the 8-peak model found by Bayesian reconstruction as the best fit for the PL data from the synthetic sample.

TABLE III. Peak-independent parameters from Bayesian reconstruction of synthetic Cu_2O PL spectrum

E_g (eV)	R_y (meV)
2.174	93.4

TABLE IV. Peak-dependent parameters from Bayesian reconstruction of synthetic Cu_2O PL spectrum.

n	δ_n	Γ_n (nm)	f_n (a.u.)
2	0.0577	0.8598	0.3590
3	0.0807	0.3713	0.0761
4	0.1436	0.2374	0.0984
5	0.1079	0.1948	0.0248
6	0.2151	0.2729	0.0265
7	0.2047	0.2560	0.0225
8	0.1645	0.3102	0.0243

[1] A. Konzelmann, B. Frank, and H. Giessen, Quantum confined rydberg excitons in reduced dimensions, Journal of Physics B: Atomic, Molecular and Optical Physics **53**, 024001 (2019).
[2] K. Orfanakis, S. K. Rajendran, H. Ohadi, S. Zielińska-Raczyńska, G. Czajkowski, K. Karpiński, and D. Ziemkiewicz, Quantum confined rydberg excitons in Cu_2O nanoparticles, Phys. Rev. B **103**, 245426 (2021).
[3] G. M. Kavoulakis, Y.-C. Chang, and G. Baym, Fine structure of excitons in Cu_2O, Phys. Rev. B **55**, 7593 (1997).
[4] M. Aßmann and M. Bayer, Semiconductor rydberg physics, Adv. Quantum Technol. **3**, 1900134 (2020).
[5] M. Takahata and N. Naka, Photoluminescence properties of the entire excitonic series in Cu_2O, Phys. Rev. B **98**, 195205 (2018).
